# Acute Fatty Liver of Pregnancy: A Thorough Examination of a Harmful Obstetrical Syndrome and Its Counterparts

**DOI:** 10.7759/cureus.2164

**Published:** 2018-02-06

**Authors:** Joshua Ronen, Shahzeb Shaheen, David Steinberg, Kevin R Justus

**Affiliations:** 1 Internal Medicine Resident Physician, Texas Tech University Health Sciences Center; 2 Ross University School of Medicine, California Hospital Medical Center; 3 Attending Physician In Family Medicine & Advanced Obstetrics, California Hospital Medical Center, Volunteer Faculty Physician at the Keck School of Medicine, University of Southern California; 4 Attending Physician In Maternal Fetal Medicine, Cedars-Sinai Medical Center, Voluntary Clinical Professor at the Keck School of Medicine Dept. of Ob-Gyn, University of Southern California

**Keywords:** acute fatty liver of pregnancy, steatohepatitis, hematology, coagulopathy, lchad deficiency, hepatology, hellp syndrome, preeclampsia, acute liver failure, disseminated intravascular coagulation

## Abstract

Diagnosed in one of every 20,000 deliveries, acute fatty liver of pregnancy (AFLP) was considered to be a deadly disease for many years. However, advancements in the clinical and surgical management of pregnant mothers have lead to a drastic decrease in maternal morbidity and mortality.

The corresponding case recounts a 23-year-old gravida 2 para 1 (G2P1) at 38 weeks gestational age (GA) with no relevant past medical or family medical history that presented to the emergency department (ED) with a five-day history of nausea, protracted vomiting, hypertension, and new-onset headache. Being late in the third trimester, preeclampsia was the top differential diagnosis while awaiting additional laboratory work-up. The work-up later revealed elevated liver function tests and bilirubin plus an abnormal coagulation profile with low fibrinogen. The differential was then shifted to AFLP versus hemolysis-elevated liver enzymes-low platelets (HELLP) syndrome. The patient was promptly transferred to the labor and delivery unit for close monitoring and delivery planning. Upon cervical examination, the patient was not dilated and was therefore determined to be remote from delivery. A cesarean section was performed and the mother was transferred to the intensive care unit (ICU) post-operatively to optimize management of her coagulopathy. Her abnormal laboratory studies normalized by post-operative day four and she was discharged home with her newborn.

## Introduction

**Figure 1:**
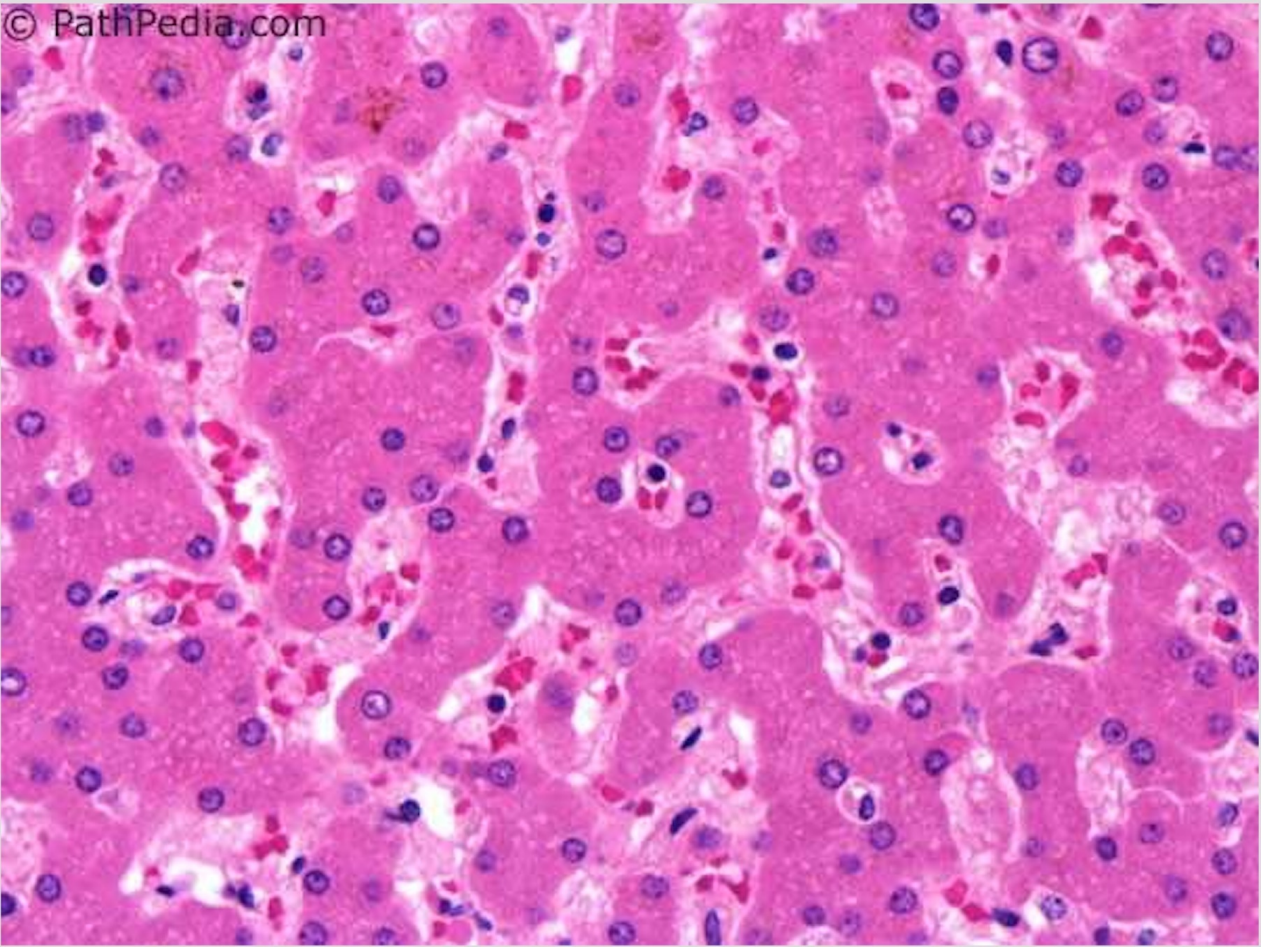
Normal Liver Histology Reference [1] This figure was used from PathPedia (online) with consent.

**Figure 2:**
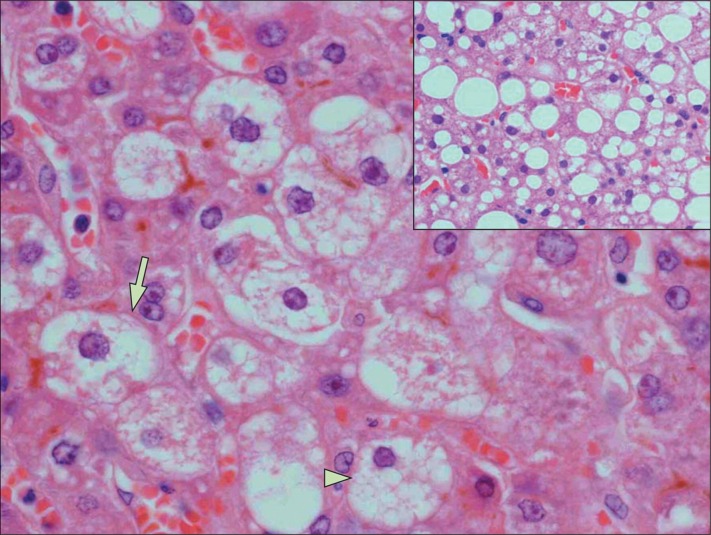
AFLP Histology Reference [2] Purple = hepatocyte nuclei. Surrounding white around nuclei = lipid droplets (steatosis). AFLP = acute fatty liver of pregnancy. This image was used from The Lancet (online) with consent.

Acute fatty liver of pregnancy (AFLP), a rare disorder unique to pregnancy, is characterized by a microvesicular fatty infiltration or steatosis of hepatocytes (see Figures 1-2) [1-2]. Prior to medical advancements and progressive research about the condition, it was initially thought to be universally fatal. Per Lee et al., AFLP is rare in the sense that it has an approximate incidence of 1/7,000 to 1/20,000 deliveries and, epidemiologically speaking, is thought to be more common in women who have multiple gestations and in women who are underweight [3]. The pathogenesis of the AFLP, in turn, has some association with inherited (heterozygous or homozygous) defects in the mitochondrial beta-oxidation of fatty acids, specifically small chain acyl CoA dehydrogenase (SCAD), medium chain acyl CoA dehydrogenase (MCAD), and most commonly, long-chain hydroxyacyl-CoA dehydrogenase (LCHAD) deficiencies. Mothers of fetuses that have inherited any of these deficiencies may be predisposed to hepatotoxicity secondary to the build of toxic substrate circulating in the maternal bloodstream. Lee et al. affirm that AFLP typically manifests itself in the third trimester of pregnancy and is thought to always present before delivery, but it is not always diagnosed as such [3]. 

## Case presentation

A 23-year-old gravida 2 para 1 (G2P1) at 38 weeks gestational age (GA) presented to the hospital emergency department (ED) with a five-day history of nausea, protracted vomiting, and poor oral intake. She sought treatment after the onset of a new severe headache. The headache lasted for four hours and was refractory to analgesic medications. The patient denied any family history of genetic disease, coagulopathies, or kidney disease. She also denied blurred vision, abdominal pain, pruritis, hematuria, spotting, or loss of fluid per vagina.

Physical exam findings were unremarkable. Vitals upon admission revealed tachycardia (137 bpm), hypertension (systolic max=148 mmHg; diastolic max=114 mmHg), and tachypnea. The patient's labs can be found below (Table 1). A retroperitoneal ultrasound was significant only for a slightly echogenic left kidney but was otherwise unremarkable. No other imaging studies were ordered.

**Table 1:** Laboratory Results Acroynms expanded: aPTT = Activated Partial Thromboplastin Time. PT = Prothrombin Time. INR = International Normalized Ratio. ALT = Alanine Aminotransferase. AST = Aspartate Aminotransferase. ALK-P = Alkaline Phosphatase. LDH = Lactate Dehydrogenase. Others: DIC = Disseminated Intravascular Coagulation. T-bili = Total Bilirubin. PLTs = Platelets. BT = Bleeding Time. AKI = Acute Kidney Injury.

Test	Actual Value	Normal Value
Glucose	47 mg/dL (L)	70-100 mg/dL
Platelets	275 x 109/L (nl)	146-429 x 109/L
Fibrinogen	61.7 mg/dL (L)	373-619 mg/dL
aPTT	49 sec (H)	24-35 sec
PT	24.2 sec (H)	9.6-12.9 sec
INR	2.2 (H)	0.8-0.94
ALT	448 U/L (H)	2-25 U/L
AST	300 U/L (H)	4-32 U/L
ALK-P	975 U/L (H)	38-229 U/L
LDH	577 U/L (H)	82-524 U/L
Total Bilirubin	10 mg/dL (H)	0.3-1.2 mg/dL
Lactic Acid	4.5 mg/dL (H)	6-16 mg/dL
Creatinine	1.8 mg/dL (H)	0.7-1.3 mg/dL
*Assessment of data = hypoglycemia, profound coagulopathy (DIC-like features), hypofibrinogenemia, transaminitis, S/S hemolysis (elevated LDH, T-bili), normal PLTs, metabolic acidosis, AKI		

**Figure 3:**
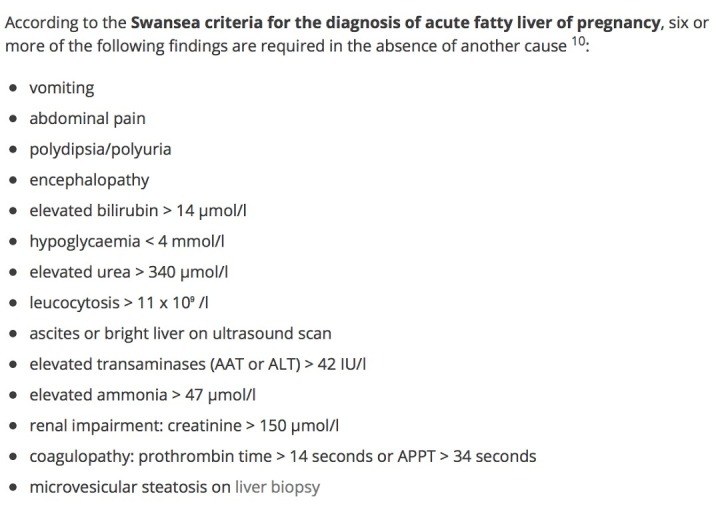
Swansea Criteria for the Dx of AFLP Reference [4]. Six criteria fulfilled: hypoglycemia, vomiting, elevated liver transaminases, renal impairment, coagulopathy, and encephalopathy. AFLP = acute fatty liver of pregnancy. This image was used from Radiopaeidia (online) with consent.

After a careful analysis of clinical and laboratory findings to rule out preeclampsia and hemolysis-elevated liver enzymes-low platelets (HELLP) syndrome (see the "Assessment of data" section at the bottom of Table 1) and an evaluation of the Swansea Criteria (Figure 3), she was given the diagnosis of AFLP [4]. The patient was made aware of her diagnosis and given the opportunity to consider her delivery options. After further clinical evaluation, a determination was made that the patient had an unfavorable cervix and was not a good candidate for a trial vaginal delivery. The patient gave informed consent and elected to undergo a cesarean section. Considering the patient’s significant coagulopathic state requiring emergent surgical intervention, she was administered a total of seven units of fresh frozen plasma (FFP) and two units of cryoprecipitate to achieve hemodynamic stability throughout her pre-, peri-, and postoperative course. She gave birth to a 2,820 gram female with an Apgar score of seven and eight at one and five minutes respectively, without experiencing any surgical complications.

After her cesarean section, the patient was transferred to the intensive care unit (ICU) where she remained for two consecutive days to optimize the management of her coagulopathy during her initial recovery. The patient experienced a rapid resolution of her symptoms and normalization of her laboratory studies during her four-day stay postpartum. By postoperative day three, vital signs and clinical studies, including liver function tests, coagulation panels, and kidney function tests, were progressively normalizing. On postoperative day four, her blood pressure was stable at 129/95 and her coagulopathy resolved. Education was provided to the patient, as it pertained to the need for genetic testing for herself and her baby, specifically screening for LCHAD deficiency. She consented to testing, which was sent out the same day prior to her discharge. Upon discharge, she was given instructions to follow up at the family medicine clinic in two days.

## Discussion

The clinical overlap between AFLP, HELLP syndrome, and preeclampsia has the potential to make these obstetrical syndromes impossible to differentiate from one another. However, per Lee et al., evidence of hepatic insufficiency, such as with hypoglycemia or encephalopathy, and abnormalities in coagulation studies are more consistent with AFLP [4]. Conversely, the most common clinical presentation of HELLP syndrome, considered to be a severe form of jaundice, is midepigastric abdominal pain and tenderness. Many patients have nausea, vomiting, and malaise that can be mistaken for a viral illness. HELLP, like AFLP, is diagnosed in the third trimester but specifically between 28-36 weeks gestation. HELLP syndrome represents a severe form of preeclampsia. Sibai et al. state that 15%-20% of patients with HELLP do not have pre-existing hypertension or proteinuria, leading some authorities to believe that HELLP is a separate disorder from preeclampsia [5]. Still, both Sibai et al. and August et al. believe preeclampsia with severe features and HELLP syndrome may be associated with serious hepatic manifestations, including infarction, hemorrhage, and rupture [5-6]. HELLP syndrome, as August et al. state, occurs on its own in <1% of pregnancies in contrast to 10%-20% of pregnancies complicated by severe preeclampsia/eclampsia syndrome [6]. Lee et al. and Kilpatrick et al. agree that signs and symptoms commonly associated with AFLP include nausea/vomiting (75% of patients), epigastric abdominal pain (50% of patients), malaise, anorexia, and jaundice [4,7-8]. Taking this evidence under consideration, it is essential to appreciate the overlap and utilize the unique qualities of these disorders to pinpoint the appropriate diagnosis in order to begin the appropriate course of management.

The patient did not have any right upper quadrant (RUQ) abdominal pain or tenderness nor thrombocytopenia (platelets: 275,000) seen commonly in HELLP syndrome. Irritation of the Glisson capsule surrounding the liver produces profuse RUQ abdominal pain in these patients. With these preceding findings in addition to the presence of hypoglycemia and hypofibrinogenemia and available evidence detailing the incidence of HELLP syndrome with or without preeclampsia, HELLP syndrome was ruled out. The patient had an uncomplicated prenatal course with normal urinalyses and blood pressure screenings despite profound coagulopathy, effectively ruling out preeclampsia subsequently yielding AFLP as our diagnosis. Due to the degree of her liver dysfunction during her hospital course, we determined that her treatment-refractory headache could have also signified the beginnings of an encephalopathic state, although other textbook sequelae of acute liver failure (ALF) were absent.

Diagnostically speaking, studies have shown that the Swansea criteria have become useful tools to screen for AFLP and to assess disease severity (Figure 3). Per Lee et al., women with AFLP can have abnormal liver function tests with alanine aminotransferase (ALT)/aspartate aminotransferase (AST), ranging from modest elevations to around 500 IU/L as well as acute kidney injury (AKI) and hyperuricemia [1,5]. Kilpatrick et al.'s findings demonstrate that clinicians can expect elevated serum bilirubin, leukocytosis (physiologic finding in pregnancy), and thrombocytopenia with or without the signs and symptoms of disseminated intravascular coagulation (DIC) [7]. DIC is also often associated with a marked reduction in antithrombin III in addition to increased bleeding time (BT), increased prothrombin time (PT)/activated partial thromboplastin time (aPTT)/international normalized ratio (INR), microangiopathic hemolytic anemia (MAHA), and schistocytes on peripheral blood smear (PBS). Lee et al. point out that severely affected patients can also have hyperammonemia and hypoglycemia secondary to hepatic insufficiency and can develop extrahepatic complications, such as infection, intra-abdominal bleeding, central diabetes insipidus (with transient polyuria and polydipsia), and severe pancreatitis (rare) [4]. A definitive diagnosis of AFLP occurs via liver biopsy but this modality is hardly used most likely due to the fact that the patient's condition would present a high risk of intraperitoneal hemorrhage due to an invasive procedure involving the extraction of tissue from the liver. A histological examination of liver biopsies, however, reveals microvesicular steatosis in Zone three (centrilobular area) of liver lobules on oil red O staining, as depicted in Figure 2 [2].

Lee et al. explain how some reports show that children born to AFLP-affected mothers have presented with sudden, unexplained death or hypoglycemia and elevated liver enzymes [4]. He and his associates highlight how infants with LCHAD, when stressed, are at risk of developing fatal nonketotic hypoglycemia imitating Reye’s syndrome, defects in the urea cycle function, neonatal dilated cardiomyopathy (DCM), and progressive neuromyopathy [4]. Lee et al., Darras et al., and Ibdah et al. each agree that utilizing genetic counselors for the purposes of testing for the known genetic variants of LCHAD with the most common and specific being a G1528C mutation (that alters amino acid 474 from glutamic acid to glutamine on protein E474Q) should be considered in all affected women with AFLP, their infants, and their fathers [4,9-10]. Darrah et al. and Ibdah et al. go further to elucidate that mutations in SCAD and MCAD, as well as in the alpha subunit of a trifunctional protein (TFP) in the active site of the LCHAD enzyme in the inner mitochondrial membrane have also been implicated [9-10].

Management

The treatment of AFLP entails a combination of maternal stabilization and prompt delivery of the fetus regardless of gestational age, as illustrated by Lee et al. [4]. Glucose infusion (10% dextrose solution, possibly additional ampoules of 50% dextrose) as well as the administration of fresh frozen plasma (FFP), cryoprecipitate, packed red blood cells (PRBCs), and platelets are given as needed. Blood glucose monitoring should be ordered for all affected patients until the normal liver function returns, as fluctuations may occur due to the liver’s role in glucose metabolism. The mode of delivery of the fetus depends on a combination of factors: fetal status, maternal status, and the probability of successful labor induction. Cesarean section is indicated if accomplishing a successful vaginal birth within 24 hours of induction is unlikely or if there is a concern for rapidly progressing maternal or fetal decompensation. In the setting of coagulopathy, delivery should be undertaken with the perioperative administration of appropriate blood products. If a patient demonstrates abnormalities in coagulation parameters outside the normal for pregnancy (third-trimester fibrinogen level<300 mg/dL, INR>1.1), especially if it is near the time of delivery, Lee et al. recommend the early administration of appropriate blood products [8]. Our patient had a fibrinogen of 61.7 and was treated per these recommendations (see Table 1).

Similarly to the management of AFLP, Sibai et al. state that the management of HELLP syndrome requires the stabilization of the mother, assessing the fetal condition, and deciding whether or not prompt delivery is indicated [5]. Severe hypertension (HTN) should be treated with antihypertensive therapy and magnesium sulfate to prevent convulsions and for neuroprotection of fetuses/neonates at 24-32 weeks of gestation. If a patient has severe complications of HELLP syndrome, such as abruptio placentae, acute renal failure (ARF), subcapsular liver hematoma, pulmonary edema, DIC, or multiorgan dysfunction, the indication is for prompt delivery regardless of gestational age - as with AFLP patients. Additionally, HELLP syndrome patients (albeit most being less than 30-32 weeks gestational age) with an unfavorable cervix should undergo a cesarean section to avoid a potentially long induction, Sibai et al. say [5]. For HELLP syndrome pregnancies >34 weeks of gestation and <23 weeks of gestation, delivery is recommended instead of expectant management. For fetuses 34 weeks GA or older, the potential risk of preterm birth outweighs the risk associated with HELLP syndrome.

The risks of AFLP include extreme susceptibility to developing coagulopathies due to the decreased liver production of coagulation factors and/or DIC, as mentioned previously. As a result, these patients are at a high risk for bleeding complications, such as postpartum hemorrhage (PPH). The patient’s platelet count, PT/PTT/INR, and fibrinogen levels should be monitored to assess for evolving or overt coagulopathy. Liver tests and coagulopathy should start to normalize after delivery usually within seven to 10 days. Liver and renal function may transiently worsen before improving postoperatively. Extreme measures to prevent maternal morbidity and mortality such as aggressive hematologic resuscitation and preterm delivery are unlikely to be needed with early diagnosis and prompt delivery. AFLP can recur in subsequent pregnancies, even if the patient's fetal LCHAD tests are negative (as in this case). The exact recurrence risk is unknown. Affected women should be warned about the possibility of recurrence in subsequent pregnancies and, as Lee et al. recommend, should be monitored by maternal fetal medicine (MFM) specialists [4].

## Conclusions

Early diagnosis and prompt delivery have become paramount in reducing unfavorable maternal outcomes in AFLP as well as the HELLP and preeclampsia/eclampsia syndromes. HELLP syndrome and preeclampsia closely mimic the clinical course of AFLP, its management, and its risks. In particular, the morbidity and mortality of AFLP patients have considerably declined in recent years due to the success of prompt delivery in producing clinical resolution. Emphasis should be placed on the early recognition and management of LCHAD and its associated enzyme deficiencies in order to curb fetal and neonatal mortality.
